# MiR-532-5p suppresses renal cancer cell proliferation by disrupting the ETS1-mediated positive feedback loop with the KRAS-NAP1L1/P-ERK axis

**DOI:** 10.1038/s41416-018-0196-5

**Published:** 2018-08-07

**Authors:** Wei Zhai, Junjie Ma, Rujian Zhu, Chen Xu, Jin Zhang, Yonghui Chen, Zhiguo Chen, Dongkui Gong, Jiayi Zheng, Chen Chen, Saiyang Li, Butang Li, Yiran Huang, Wei Xue, Junhua Zheng

**Affiliations:** 1 Department of Urology, Renji Hospital, School of Medicine in Shanghai Jiao Tong University, Shanghai, 200127 China; 2 Department of Urology, Shanghai Tenth People’s Hospital, Nanjing Medical University, Nanjing, 211166 China; 3 Department of Urology, Pudong Hospital, School of Medicine in Fudan University, Shanghai, 201300 China; 4 Department of Urology, Shanghai Tenth People’s Hospital, School of Medicine in Tongji University, Shanghai, 200072 China; 5 Department of Pathology, Shanghai Tenth People’s Hospital, School of Medicine in Tongji University, Shanghai, 200072 China; 6 Department of Urology, Shanghai First People’s Hospital, School of Medicine in Shanghai Jiao Tong University, Shanghai, 200080 China

**Keywords:** Cell growth, Tumour biomarkers, miRNAs

## Abstract

**Background:**

Despite the fact that miRNAs play pivotal roles in various human malignancies, their molecular mechanisms influencing RCC are poorly understood.

**Methods:**

The expression of miRNAs from RCC and paired normal renal specimens was analysed by a combined computational and experimental approach using two published datasets and qRT-PCR assays. The functional role of these miRNAs was further identified by overexpression and inhibition assays in vivo and in vitro. Western blots, luciferase assays, and chromatin immunoprecipitation were performed to investigate the potential mechanisms of these miRNAs.

**Results:**

Bioinformatics analysis and qRT-PCR revealed that miR-532-5p was one of the most heavily downregulated miRNAs. Overexpression of miR-532-5p inhibited RCC cell proliferation, while knockdown of miR-532-5p promoted cell proliferation. Mechanistic analyses indicated that miR-532-5p directly targets KRAS and NAP1L1. Interestingly, ETS1 suppressed the transcription of miR-532-5p by directly binding a special region of its promoter. Moreover, high levels of ETS1, as an oncogene in RCC, were significantly associated with poor survival in a large cohort of RCC specimens.

**Conclusions:**

Our work presents a road map for the prediction and validation of a miR-532-5p/KRAS-NAP1L1/P-ERK/ETS1 axis feedback loop regulating cell proliferation, which could potentially provide better therapeutic avenues for treating RCC.

## Introduction

Renal cell carcinoma (RCC) is one of the most common and aggressive human malignancies throughout the world, representing 3.7% of all new cancer cases, with about 65,340 new cases and 14,970 deaths estimated for 2018 in the United States alone.^1,2^ In most cases, RCC is radiotherapy and chemotherapy resistant and best treated by surgical resection.^3–5^ Although tyrosine kinase inhibitor (TKI)-based antiangiogenic therapy is the standard treatment for advanced RCC (aRCC) or metastatic RCC (mRCC), the overall prognosis remains poor due to drug resistance and other reasons.^2,6^ Therefore, methods for earlier diagnosis and more effective therapies for RCC are urgently required.

MicroRNAs (miRNAs), a class of small noncoding RNAs with transcripts averaging 22 nucleotides in length, negatively regulate target gene expression through mRNA degradation or translational repression.^7^ Recently, miRNAs have emerged as key molecules that contribute to cellular proliferation, migration, invasion, and metastasis in numerous human cancers, including RCC.^8–11^ Several miRNAs have been confirmed to inhibit tumour progression in RCC, such as miR-646 and miR-766-3p, which was previously demonstrated by our group.^10,11^ To date, a growing body of evidence indicates that miR-532-5p serves as a tumour promoter or suppressor in multiple human cancers.^12,13^ However, the biological role of miR-532-5p in RCC has been unknown to this point.

The mitogen-activated protein kinases/extracellular signal-regulated kinase (MAPK/ERK) pathway has been shown to be regulated by various miRNAs and proteins, and is involved in cell proliferation, migration, and chemotherapeutic therapy resistance.^8,14,15^ KRAS, a Kirsten ras oncogene homolog from the mammalian ras gene family, potentially influences multiple facets of malignant transformation.^16,17^ Recent studies have elucidated its biological effects in various cancers, which include activating downstream-signalling pathways, such as the RAF-MEK-ERK cascade and enhancing cell proliferation.^18,19^ NAP1L1 (nucleosome assembly protein 1-like 1), which is highly conserved compared with other genes from the Nap1 family, is involved in nucleosome assembly, cell cycle progression, and cell proliferation.^20^ Interestingly, NAP1L1 displays higher nucleosome disassembly activity.^21^ Previous reports identified that NAP1L1 was highly expressed in some tumour tissues, such as hepatoblastomas^22^ and small intestinal carcinoids.^23^ In addition, it is involved in AKT and ERK signalling in induced pluripotent stem cells (iPSC).^24^ However, little is known regarding its expression and biological role in RCC.

ETS1 encodes a member of the ETS family of transcription factors and is involved in cancer progression.^25,26^ To date, ETS family members have been shown to participate in cell proliferation and differentiation and do so through recognising a GCA core sequence in the promoter or enhancer of target genes.^27^ Previous studies have reported that ETS1 could be driven by MAPK signalling and modulates the expression of miRNAs in lung squamous cell carcinomas (SCC) with an immune evasion subtype.^14^ However, the cross-talk between ETS1 and miRNAs in RCC is still unknown.

Here, we investigated the role of miR-532-5p, a novel tumour suppressor that repressed RCC tumourigenesis. Moreover, we demonstrated the relationship between miR-532-5p, KRAS, NAP1L1, and ETS1 in RCC and found an atypical miR-532-5p/KRAS-NAP1L1/P-ERK/ETS1 feedback loop. In summary, our study presented a novel mechanism by which miR-532-5p inhibited RCC cell growth.

## Methods

### Microarray analysis and TCGA dataset analysis

Microarray analysis for the expression of miRNAs and TCGA data set analysis was performed by the Shanghai Gminix Biological Information Company (Shanghai, China). The microarray data used in this paper were downloaded from the Gene Expression Omnibus database GEO (https://www.ncbi.nlm. nih.gov/geo/), and the accession numbers were GSE41282 and GSE23085.^28,29^ The preprocessed level-3 RNA-seq data and the corresponding clinical information of renal cell carcinoma (RCC) patients were collected from the Cancer Genome Atlas (TCGA) database (http://cancergenome.nih.gov/). We used the edgeR package of R packages to perform the difference analysis (http://www.bioconductor.org/packages/release/bioc/html/edgeR.html) and used the pheatmap package of R packages to perform the cluster analysis (https://cran.r-project.org/web/packages/pheatmap/index.html). Sva R package was used to remove the batch effect. MiRNAs/genes with adjusted *p* values < 0.05 and absolute fold changes (FC) > 1.5 were considered differentially expressed miRNAs/genes. Kaplan–Meier survival curves were drawn to analyse the relationships between miRNAs/genes and overall survival in the survival package. We used a Pearson *χ*^2^ test to examine the association of miRNAs with genes. The corresponding statistical analysis and graphics were performed in R software (R version 3.3.2).

### Human specimens

All surgical specimens (paired normal and tumour tissues) were collected from patients with RCC in the Shanghai Tenth People’s Hospital, Tongji University School of Medicine (China). All fresh samples were immediately preserved in liquid nitrogen to protect the protein or RNA from degradation. This study was approved by the Shanghai Tenth People’s hospital ethics committee and written informed consent was obtained from all patients.

### Cell cultures

The human RCC cell lines 786-O, OSRC-2, A498, and human normal renal tubular epithelial cell line HK-2 were obtained from the Cell Bank of the Chinese Academy of Sciences (Shanghai, China). The SN12-PM6 cell line was kindly provided by Dr. Qingbo Huang from the Department of Urology, Chinese PLA General Hospital, Beijing, China. A total of 786-O and OSRC-2 cells were cultured in RMPI 1640 (Gibco, USA) plus 10% foetal bovine serum (FBS, Hyclone, USA) with 1% penicillin/streptomycin (P/S, Gibco, USA). A498 and SN12-PM6 cells were cultured in Dulbecco’s modified Eagle’s medium (DMEM, Gibco, USA) plus 10% foetal bovine serum (FBS, Hyclone, USA) with 1% penicillin/streptomycin (P/S, Gibco, USA). HK-2 cells were cultured in keratinocyte medium (KM, ScienCell, USA) plus 1% keratinocyte growth supplement (KGS, ScienCell, USA) with 1% penicillin/streptomycin (P/S, ScienCell, USA). All cell lines were incubated in a humidified chamber at 37 °C in 5% CO_2_.

### Cell transfection and vector construction

According to the manufacturer’s instructions, miR-532-5p/miR-NC (GenePharma, China), anti-miR-532-5p/anti-miR-NC (GenePharma, China), short interfering RNA (siRNA) si-KRAS/si-NAP1L1/siRNA-NC (Genepharma, China), and locked nucleic acid miRNA (LNA-miRNA) and LNA-miR-532-5p/LNA-miR-NC (IBS Solutions Co. Ltd, China) were transiently transfected in SN12-PM6 or 786-O cells using Lipofectamine 3000 (Invitrogen, USA) at a final concentration of 50 nM. pWPI-oe-ETS1 and negative control vector were transiently transfected in 786-O cells by Lipofectamine 3000 for a luciferase assay. The cells were harvested for subsequent experiments 48 h after transfection. Lentiviral miR-532-5p/miR-NC and sh-miR-532-5p/sh-miR-NC were purchased from Ling ke Biotechnology (Shanghai, China). Lentiviral infection was performed as previously described.^28,29^ The sequences of oligonucleotides used in this study are listed in Supplementary Table S1.

### CCK8, colony formation, and fluorescence-activated cell-sorting assays

The Cell Counting Kit-8 (CCK-8, Dojindo, China) was used to measure cell proliferation according to the manufacturer’s protocol. Cells were seeded into 96-well plates at a density of 1 × 10^3^ cells per well and cultured for 24, 48, 72, 96, or 120 h. After incubation for 2 h with CCK-8 (10 μl) at 37 °C in 5% CO_2_, the optical density (OD) was measured at 450 nm by an auto-microplate reader (BioTek, USA). For colony formation assays, transfected cells were seeded in a six-well plate at a density of 6 × 10^2^ cells per well. After culturing for 10 days, the colonies were washed twice with PBS, fixed in 95% alcohol, and stained with 0.1% crystal violet solution. For the cell cycle analysis, the transfected cells were collected and fixed with 75% alcohol over 4 h at –20 °C. Fluorescence-activated cell-sorting (FACS; BD Biosciences) analysis was performed using propidium iodide stains for cell cycle analysis according to the manufacturer’s protocol.

### RNA isolation and quantitative real-time PCR

Total RNA was extracted from frozen tissues or cultured cells using Trizol reagent (Invitrogen, USA), according to the manufacturer’s instructions. The concentration and purity of RNA was determined using an ND-2000 Spectrophotometer (Thermo Fisher Scientific, USA). For miR-532-5p level detection, complementary DNA synthesis was performed using a PrimeScript RT reagent kit (TaKaRa, Japan), and quantitative real-time PCR (qRT-PCR) was performed with the KAPA SYBR FAST qPCR Kit (Kapa Biosystems, USA) using a 7900HT Fast Real-Time PCR System (Applied Biosystems, Japan). The expression levels of miR-532-5p were normalised to endogenous small nuclear RNA U6. Data were analysed using the 2^–ΔΔCt^ method. The primer sequences are listed in Supplementary Table S1. The specificity of amplification products was confirmed by melting-curve analysis.^30^

### Western blot analysis

Protein extracts were separated from cells or human tissues with RIPA buffer containing protease inhibitors. Next, 30 µg of protein extract was loaded onto 8–10% sodium dodecylsulfate–polyacrylamide gel electrophoresis gels and transferred onto nitrocellulose membranes. The membranes were hybridised with a primary antibody at 4°C overnight and incubated with a secondary antibody for 1 h at room temperature. The expression of β-actin was used as a loading control. The protein signal was visualised using an Odyssey scanner (LI-COR Biosciences, USA). The antibodies used were as follows: KRAS (1:500, Abcam, ab180772), NAP1L1 (1:1000, Abcam, ab33076), ETS1 (1:1000, CST, D8O8A), ERK1 + ERK2 (1:10,000, Abcam, ab184699), phospho-ERK1 + phospho-ERK2 (1:1000, Abcam, ab201015), Ki67 (1:5000, Abcam, ab92742), and β-actin (1:1000, Abcam, ab8226).

### Luciferase assay

A luciferase reporter assay was performed as previously described.^31^ The 3’-UTR of KRAS or NAP1L1 was constructed into a pWPI-LUC vector (Promega, USA), and promoter regions of miR-532-5p–5p were constructed into a pGL3-basic vector (Promega, USA). According to the manufacturer’s manuals, cells were cultured in 24-well plates, and the cDNA, miRNA-532-5p mimic, or miR-NC was transfected by Lipofectamine 3000 (Invitrogen, USA). pRL-TK was used as an internal control. According to the manufacturer’s instruction, luciferase activity was measured using the dual-luciferase assay reagent (Promega, USA).

### Chromatin immunoprecipitation assay

Cells were cross-linked with 1% formaldehyde to covalently cross-link proteins to DNA, followed by chromatin collection. Cross-linked DNA was sheared into 300–500-bp long fragments via sonication. Lysates were precleared sequentially with normal rabbit IgG (sc-2027, Santa Cruz Biotechnology) and protein A agarose. An anti-ETS1 antibody (2.0 µg) was added to the cell lysates and incubated at 4 °C overnight. IgG was used in the reaction as the negative control. Finally, PCR was used to measure the enrichment of DNA fragments in the putative ETS1-binding sites in the miR-532-5p promoter using specific primers (Table S1). PCR products were analysed by agarose gel electrophoresis.

### Mouse model of xenograft subcutaneous implantation and orthotopic tumour implantation

All BALB/c nude mice were purchased from the Shanghai Sipper-BK laboratory animal Company (Shanghai, China). Briefly, a total of 4 × 10^6^ miR-532-5p stably overexpressing or miR-NC 786-O cells were implanted hypodermically into the right oxter of 4-week-old nude mice. The tumour volume and the weight of each mouse was measured each week, and mice were killed 8 weeks after injection. Luciferase stably expressing SN12-PM6 cells with sh-miR-532-5p or sh-miR-NC (at 2 × 10^6^, mixed with Matrigel, 1:1) was injected into the left subrenal capsule of 5-week-old male nude mice orthotopically. Primary lesions were monitored using an in vivo imaging system (IVIS) (NightOWL II, LB983, Berthold Technologies, Germany) once a week. Eight weeks after injection, animals were killed. All primary tumours and metastases isolated from mice were used for immunohistochemical staining or histological staining with haematoxylin and eosin (H&E). All animal studies were approved by the Institutional Animal Care and Use Committee of the Shanghai Tenth People’s Hospital.

### Immunohistochemistry

Immunohistochemistry (IHC) staining was performed as previously described^8^ with antibodies specific for KRAS (1:100, Abcam, ab180772), NAP1L1 (1:100, Abcam, ab33076), ETS1 (1:100, CST, D8O8A), and Ki67 (1:500, Abcam, ab92742). Immunohistochemically stained tissue sections were assessed separately by at least two pathologists. Three high-power fields (magnification, ×400) were randomly selected from renal cancer tissues and normal renal tissues for histological scoring. The positive degree was classified according to scoring both the proportion of positive-staining tumour cells and the staining intensities. Scores representing the proportion of positively stained tumour cells were graded as 0 (<10%), 1 (11–25%), 2 (26–50%), 3 (51–75%), and 4 (>75%). The intensity of staining was determined as 0 (no staining), 1 (weak staining = light yellow), 2 (moderate staining = yellow brown), and 3 (strong staining = brown). The staining index (SI) was calculated as the product of staining intensity × the percentage of positive tumour cells, resulting in scores of 0, 1, 2, 3, 4, 6, 8, 9, and 12. Only cells with a clear tumour cell morphology were scored.

### Gene set enrichment analysis

We used GSEA v2.0 to perform GSEA on various gene signatures. Gene sets were obtained either from the MSigDB database v4.0 or from published gene signatures. Statistical significance was assessed by comparing the enrichment score to enrichment results generated from 1000 random permutations of the gene set to obtain *p* values (nominal *p* value).

### Statistical analysis

Statistical analyses were performed using R software (R version 3.3.2), GraphPad Prism Software (7.0), and the SPSS 17.0 statistical software package (IBM, USA). One-way ANOVA, LSD *t* test, log-rank test, Pearson *χ*^2^ test, and Cox regression analyses were performed for comparisons. *p* < 0.05 was considered significant.

## Results

### miR-532-5p is downregulated in RCC tissues and cell lines

**Fig. 1:**
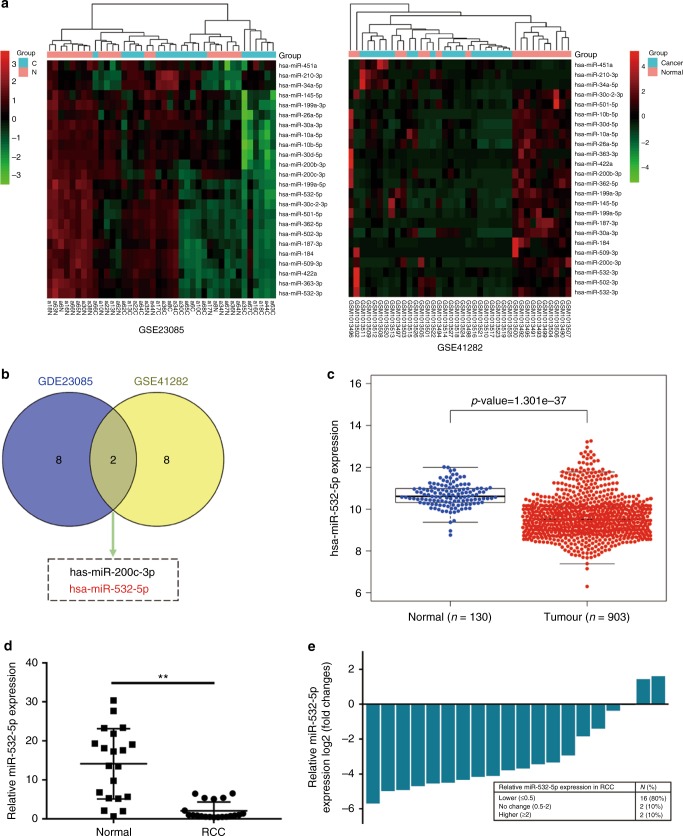

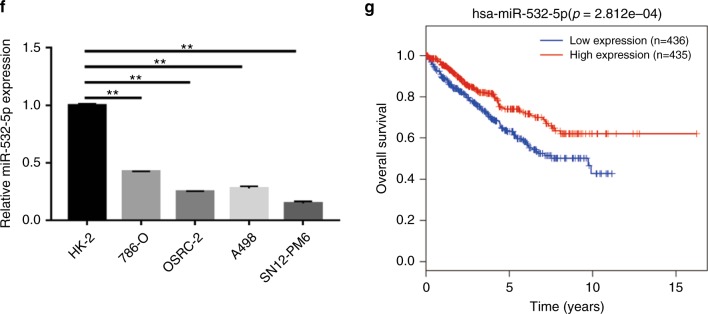
miR-532-5p is downregulated in RCC tissues and cell lines. **a** Heat map of joint analysis of differentially expressed miRNAs from two RCC miRNA arrays and miRNAs differentially expressed in both miRNA arrays. **b** The overlap of top ten downregulated miRNAs from each miRNA array. **c** miR-532-5p expression in RCC and normal samples from the TCGA RCC data set. **d** The expression of miR-532-5p was determined in tumour tissues and their adjacent non-cancer tissues by qRT-PCR. U6 was used as an internal control. **e** Relative miR-532-5p expression levels in RCC tumour are presented as fold change = 2^(ΔCt normal–ΔCt tumour)^ of tumour versus matched normal tissues. The 0.5-fold change threshold was defined as differentially expressed. **f** The expression of miR-532-5p was determined in several human RCC cell lines (786-O, OSRC-2, A498, and SN12-PM6) and human normal renal tubular epithelial cell line HK-2. **g** Kaplan–Meier analyses of the correlations between miR-532-5p expression and overall survival of 871 RCC patients from the TCGA RCC data set. Log-rank test was used to calculate *p* values

To explore differentially expressed miRNAs and their functions in RCC, we first performed joint analysis of two miRNA arrays (GSE41282 and GSE23085) from GEO data sets, comparing RCC tissues with paired normal tissues. Aberrantly expressed miRNAs are listed in Fig. 1a. Next, we selected the top ten downregulated miRNAs and found that only two miRNAs (miR-200c-3p and miR-532-5p) overlapped (Fig. 1b, Table S2 and S3). Given that miR-200c-3p has already been reported to play a role in RCC,^32^ we selected miR-532-5p as the potential candidate in our study, which was not previously investigated in RCC.

To determine the expression levels of miR-532-5p in RCC, we analysed the RCC data set from the TCGA database and found that the transcriptional level of miR-532-5p was significantly downregulated in RCC tissue compared with normal renal tissue (Fig. 1c, Table S4). In addition, we selected 20 RCC patients and examined the miR-532-5p expression (using qRT-PCR) in renal tumours and paired noncancerous tissues after operation. In agreement with other findings, the expression of miR-532-5p was significantly lower in 80% (16/20) of RCC tissues than in the paired noncancerous renal tissues (*p* < 0.01) (Fig. 1d, e). We further examined the different expression levels of miR-532-5p in four RCC cell lines (786-O, OSRC-2, A498, and SN12-PM6), as well as in a human normal renal tubular epithelial cell line HK-2 and found that the expression of miR-532-5p was dramatically decreased in RCC cell lines compared with HK-2 (Fig. 1f). In addition, we explored the association between miR-532-5p expression and RCC clinical–pathological characteristics. Correlation regression analysis of 83 samples from the Urology Department of Shanghai Tenth People’s Hospital again demonstrated that low expression of miR-532-5p was clearly associated with tumour size (*p* = 0.007), T stage (*p* = 0.022), and Fuhrman grade (*p* = 0.012) (Table S5). At the same time, we used univariate and multivariable logistic regression models to analyse the correlation of the miR-532-5p level with overall survival of 233 RCC patients. Patient characteristics can be reviewed in Table S6. Univariate analysis revealed that a higher level of miR-532-5p expression (hazard ratio, HR = 0.323; 95% confidence interval, CI = 0.018–0.639; *p* = 0.001), a larger tumour size (HR = 1.599; 95% CI = 1.297–4.209; *p* = 0.013), and a higher clinical T stage (HR = 1.965; 95% CI = 1.533–3.746; *p* = 0.026) were potently correlated with overall survival. Multivariate analysis showed that a higher miR-532-5p expression level (HR = 0.702; 95% CI = 0.339–0.953; *p* = 0.036) was markedly associated with overall survival (Table S6). Subsequently, Kaplan–Meier survival analysis from TCGA RCC datasets suggested that RCC patients with high miR-532-5p expression had longer survival times than patients with low miR-532-5p levels (log-rank test, *p* < 0.001, Fig. 1g, Table S7). Collectively, these results indicate that higher levels of miR-532-5p expression might be predictive of a better prognosis in RCC.

### miR-532-5p inhibits renal cancer cell proliferation in vitro

**Fig. 2:**
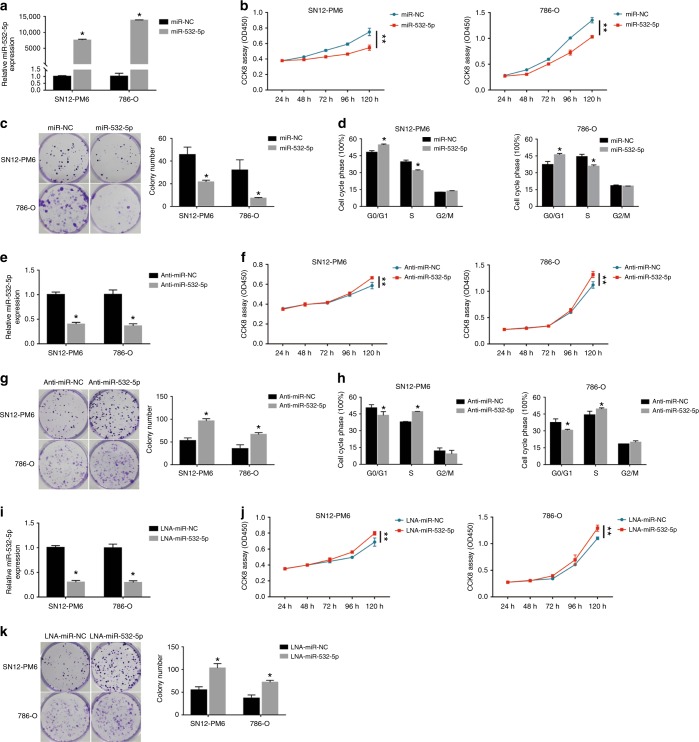
miR-532-5p inhibits renal cancer cell proliferation in vitro. **a** The expression of miR-532-5p was determined by qRT-PCR in SN12-PM6 and 786-O cells after transfection with the miR-532-5p mimic or miR-NC. **b** Cell proliferation was analyzed by CCK8 assays in SN12-PM6 and 786-O cells after transfection with the miR-532-5p mimic or miR-NC. **c** Colony formation was determined in SN12-PM6 and 786-O cells after transfection with the miR-532-5p mimic or miR-NC. The results were averaged from three experiments; error bars indicate ± 1 SD, *p < 0.05, **p < 0.01. **d** Cell cycle analysis by flow cytometer assays in SN12-PM6 and 786-O cells after transfection with miR-NC or miR-532-5p. **e** The expression of miR-532-5p was determined by qRT-PCR in SN12-PM6 and 786-O cells after transfection with anti-miR-532-5p or anti-miR-NC. **f** Cell proliferation was analyzed by CCK8 assays in SN12-PM6 and 786-O cells after transfection with anti-miR-532-5p or anti-miR-NC. **g** Colony formation was determined in SN12-PM6 and 786-O cells after transfection with anti-miR-532-5p or anti-miR-NC. The results were averaged from three experiments; error bars indicate ± 1 SD, *p < 0.05, **p < 0.01. **h** Cell cycle analysis by flow cytometer assays in SN12-PM6 and 786-O cells after transfection with anti-miR-NC or anti-miR-532-5p. **i** The expression of miR-532-5p was determined by qRT-PCR in SN12-PM6 and 786-O cells after transfection with LNA-miR-532-5p or LNA-miR-NC. **j** Cell proliferation was analyzed by CCK8 assays in SN12-PM6 and 786-O cells after transfection with LNA-miR-532-5p or LNA-miR-NC. **k** Colony formation was determined in SN12-PM6 and 786-O cells after transfection with LNA-miR-532-5p or LNA-miR-NC. The results were averaged from three experiments; error bars indicate ± 1 SD, *p < 0.05, **p < 0.01

To further identify and validate the role of miR-532-5p in RCC development, we selected the 786-O cell line with relatively high expression levels of miR-532-5p and the SN12-PM6 cell line with relatively low expression levels for subsequent experiments (Fig. 1f). MiR-532-5p mimics or miR-NC were transfected into SN12-PM6 and 786-O cells. Using qRT-PCR, we confirmed that the expression of miR-532-5p was upregulated in SN12-PM6 and 786-O cells after the transfection with the miR-532-5p mimic compared with cells transfected with miR-NC (Fig. 2a). CCK8 assays showed that upregulation of miR-532-5p suppressed RCC cell proliferation in both SN12-PM6 and 786-O cell lines compared with a negative control group (Fig. 2b). Similarly, colony formation assays also indicated that overexpression of miR-532-5p inhibited cell growth in both cell lines (Fig. 2c). Furthermore, miR-532-5p significantly enhanced the percentage of cells in the G0/G1 phase, while it reduced the percentage of cells in the S phase, which was determined by flow cytometry, for both cell lines (Fig. 2d and S1A). By contrast, we confirmed that the expression of miR-532-5p was downregulated in SN12-PM6 and 786-O cells after transfection with the miR-532-5p inhibitor (anti-miR-532-5p), compared with cells transfected with anti-miR-NC (Fig. 2e). To confirm our results, we used another miR-532-5p inhibitor (LNA-miR-532-5p) and miR-532-5p expression was detected in both cell lines (Fig. 2i). CCK8 assays, colony formation assays, and FACS results all showed that knocking down miR-532-5p expression promotes RCC cell growth and increases the percentage of cells in the S phase (Fig. 2e–k and S1B). Altogether, our data demonstrate that miR-532-5p functions as a tumour suppressor in RCC cell lines.

### miR-532-5p directly regulates KRAS and NAP1L1

**Fig. 3:**
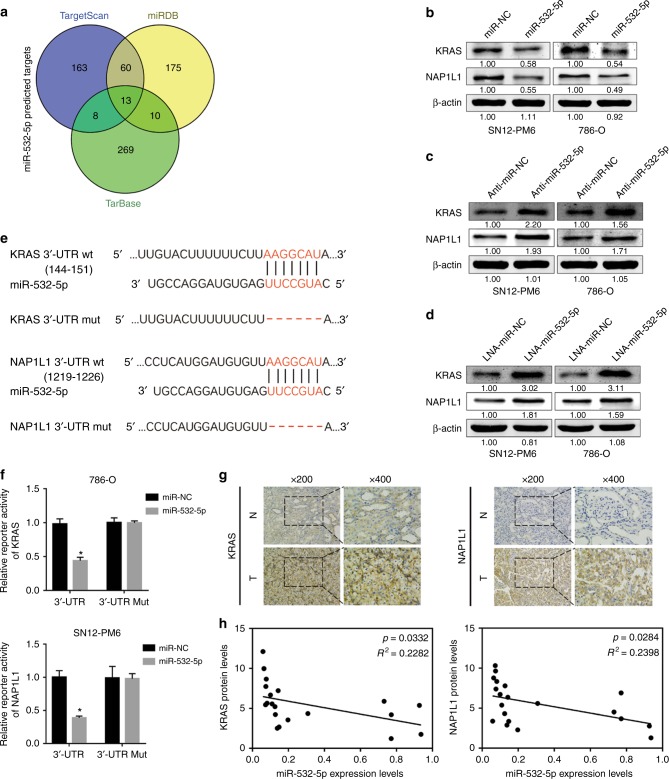
miR-532-5p directly regulates KRAS and NAP1L1. **a** Venn diagram depicting the overlap of potential target genes of miR-532-5p. **b**, **c**, **d** Western blots of KRAS and NAP1L1 expression in miR-532-5p-overexpressing or miR-532-5p-inhibited SN12-PM6 and 786-O cells, β-actin was used as a loading control. **e** miR-532-5p and its putative binding sequences in the 3’-UTR of KRAS and NAP1L1. Two mutations were generated in the complementary site that binds to the seed region of miR-532-5p. **f** Luciferase reporter assays of KRAS and NAP1L1 3’-UTRs in 786-O or SN12-PM6 cells; error bars indicate ± 1 SD, *p < 0.05, **p < 0.01. **g** Representative KRAS and NAP1L1 IHC staining of RCC tissues compared to paired normal renal tissues (200×, 400×). **h** The correlation between miR-52-5p expression and KRAS/NAP1L1 protein levels in 20 RCC tissues

To further identify the mechanism underlying miR-532-5p-modulated suppression of RCC proliferation, we sought potential downstream target genes of miR-532-5p through three different miRNA target-predicting algorithms (TargetScan, TarBase, and miRDB) and then focused on potential oncogenes associated with cell proliferation from 13 candidate target genes (Fig. 3a and Figure S1C).^17,33^ Western blot analysis consistently showed that after transfection of the miR-532-5p mimic or the miR-532-5p inhibitor, the protein levels of KRAS and NAP1L1 were reduced in miR-532-5p mimic-treated RCC cells (Fig. 3b), while they were elevated in cells where miR-532-5p inhibition was performed (Fig. 3c, d). Notably, WB assays also suggested that KRAS and NAP1L1 protein levels were increased in RCC cell lines compared to HK-2 cells (Figure S1D). Next, using a computational prediction of miRNA databases (TargetScan), we found a putative binding site for miR-532-5p with high complementarity in the KRAS 3’ untranslated region (UTR) and the NAP1L1 3’-UTR. Thus, we constructed luciferase reporters by cloning the 3’-UTR of KRAS wild-type (wt) and KRAS mutant-type (mut) with a deletion complementary sequence, as well as the 3’-UTR of NAP1L1 wt and NAP1L1 mut (Fig. 3e). Dual-luciferase reporter assays revealed that luciferase activity associated with KRAS and NAP1L1 wt 3’-UTR, but not the mut 3’-UTR, was repressed in miR-532-5p mimic-transfected 786-O or SN12-PM6 cells, compared with control cells (Fig. 3f). Subsequently, IHC analysis demonstrated that KRAS and NAP1L1 protein levels were significantly higher in RCC tissues than in their noncancerous counterparts (Fig. 3g). We went on to examine the correlation of KRAS and NAP1L1 with miR-532-5p in 20 renal cancer tissues. As we expected, the miR-532-5p transcriptional level was negatively associated with KRAS and NAP1L1 protein levels (Fig. 3h). In addition, TCGA RCC datasets also showed that NAP1L1 expression in RCC tissue was higher than in normal tissue, and the correlation between NAP1L1 and miR-532-5p was negative (Figure S1E and S1F, Table S8 and S9). Moreover, Kaplan–Meier survival analysis from the TCGA RCC dataset showed that patients with lower NAP1L1 expression levels had longer survival times than those with higher NAP1L1 expression levels (Figure S1G, Table S10). Collectively, these results suggested that miR-532-5p suppresses KRAS and NAP1L1 expression and is negatively related with its target genes in RCC cells.

### miR-532-5p attenuates the growth of RCC cells via the KRAS-NAP1L1/P-ERK/ETS1 signalling pathway

**Fig. 4:**
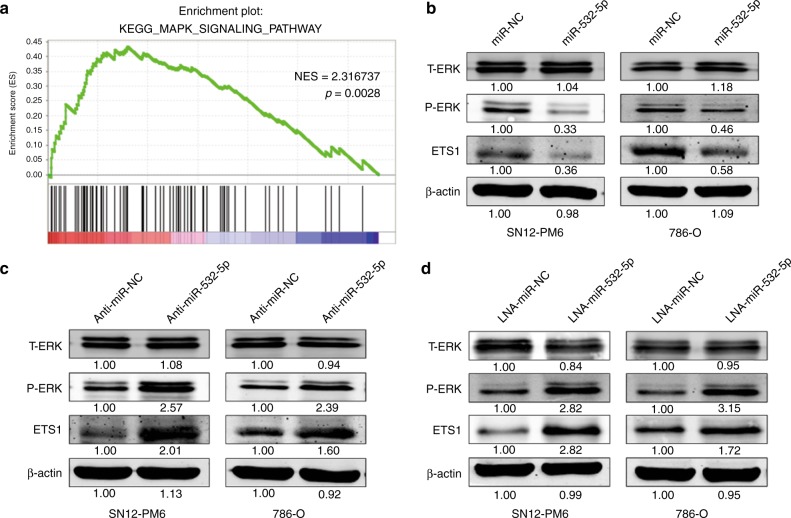
miR-532-5p attenuates the growth of RCC cells via the KRAS-NAP1L1/P-ERK/ETS1 signalling pathway. **a** GSEA of MAPK-signalling pathway signatures in published miRNA arrays. **b**, **c**, **d** Expression of T-ERK, P-ERK, and ETS1 was detected following 50 nM transfection of miR-532-5p mimics or inhibitors (anti-miR-532-5p or LNA-miR-532-5p) compared to NC (miR-NC, anti-miR-NC, and LNA-miR-NC) in SN12-PM6 and 786-O cells. β-actin was used as a loading control

Previous studies demonstrated that both KRAS and NAP1L1 promoted tumourigenesis by regulating MAPK pathway activity.^19,24,34^ To elucidate whether miR-532-5p plays a role in MAPK pathway signalling, we first performed gene set enrichment analysis (GSEA) to link the published gene array analysis of different-stage RCCs and matched normal kidney tissue signatures (GEO Datasets: GSE6344).^35^ GSEA supported that the MAPK-signalling pathway was significantly enriched in the RCC group, strongly suggesting that RCC is closely related to the MAPK-signalling pathway (Fig. 4a). To investigate whether the MAPK pathway associated protein was involved in miR-532-5p-KRAS/NAP1L1-modulated cell proliferation in RCC, we performed WB analysis, which showed that miR-532-5p could blunt KRAS-NAP1L1/P-ERK signalling in both SN12-PM6 and 786-O cell lines (Fig. 4b). Concomitant with a decrease in P-ERK in MAPK signalling, we observed a decrease in ETS1 levels (Fig. 4b), which coincided with a well-established role of ETS1 as an effector of MAPK signalling.^14,36,37^ On the contrary, anti-miR-532-5p and LNA-miR-532-5p could enhance KRAS-NAP1L1/P-ERK/ETS1 signalling in both cell lines (Fig. 4c, d). Altogether, our data suggested that miR-532-5p could attenuate RCC proliferation through modulation of KRAS-NAP1L1/P-ERK/ETS1 signalling.

### ETS1 inhibits miR-532-5p by binding to its promoter region and is associated with a poor prognosis in RCC

**Fig. 5:**
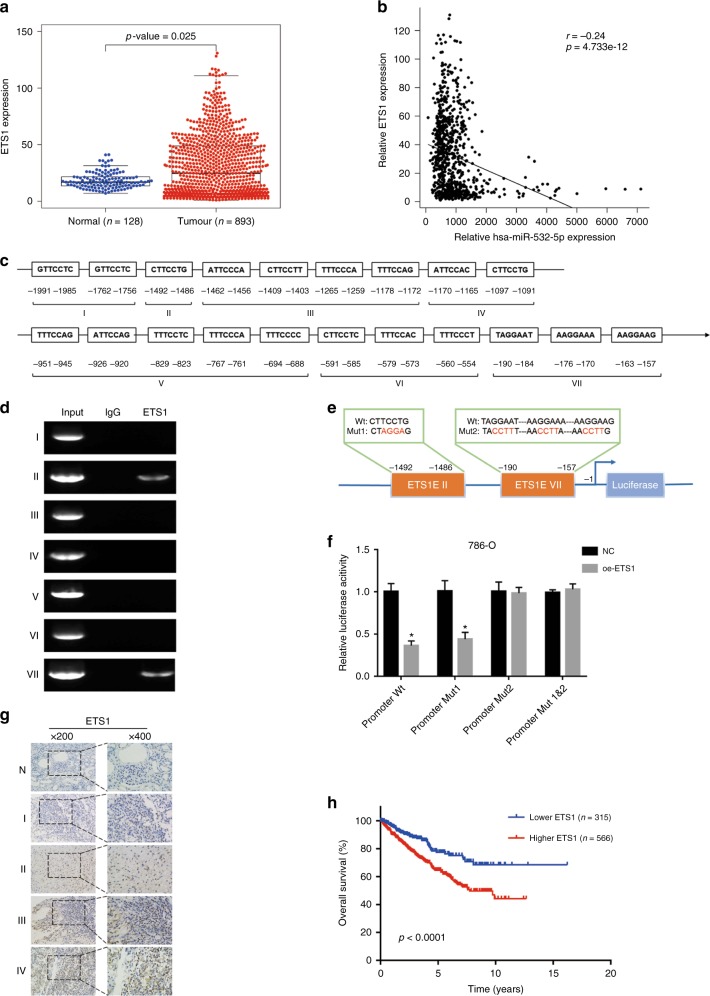
ETS1 inhibits miR-532-5p by binding to its promoter region and is associated with a poor prognosis in RCC. **a** ETS1 expression in RCC and normal samples from the TCGA RCC data set. **b** TCGA RCC dataset indicated the correlation between ETS1 and miR-532-5p in RCC. **c** Bioinformatics analysis of potential ETS1-binding sites in the miR-532-5p promoter using an online software ALGGEN PROMO. **d** ChIP assays showing that ETS1 can bind to potential binding sites in the miR-532-5p promoter. **e** A schematic illustration of ETS1E II-mut and ETS1E VII-mut in the miR-532-5p promoter. **f** Relative luciferase activity of the indicated promoter vectors in 786-O cells transfected with Renilla luciferase plasmids. **g** Representative ETS1 IHC staining of RCC tissues with different clinical stages compared to normal renal tissues (200×, 400×). **h** Kaplan–Meier analyses of the correlation between ETS1 expression and overall survival of 881 RCC patients from the TCGA RCC data set. Log-rank test was used to calculate *p* values

Accumulating evidence indicates that ETS1, acting as a transcriptional factor, positively or negatively regulates numerous miRNAs and takes part in tumour progression in various human cancers.^14,36,37^ To explore the functional role of ETS1 in RCC, we analysed the RCC dataset from the TCGA database and found that the expression of ETS1 was saliently increased in RCC tissues compared with normal renal tissues (Fig. 5a, Table S11). Intriguingly, the correlation analysis from the TCGA RCC dataset also suggested that ETS1 expression was negatively related with the expression of miR-532-5p (Fig. 5b, Table S12). Based on the data above, we hypothesised that ETS1 could directly decrease miR-532-5p expression by binding to potential ETS1 elements (ETS1Es) in the promoter. PROMO bioinformatics software was utilised to analyse 2000 bases upstream of the transcriptional start site (TSS) of the precursor miR-532-5p, pre-miR-532-5p, and potential ETS1Es upstream from the TSS. Seven ETS1-binding motifs were identified inside the putative miR-532-5p promoter region. These transcription factor-binding sites were named I, II, III, IV, V, VI, and VII (Fig. 5c). A chromatin immunoprecipitation assay (ChIP) assay with an anti-ETS1 antibody confirmed that ETS1 could bind to the ETS1Es located between the ETS1E II (–1492 to –1486) and ETS1E VII (–190 to –157) nucleotides of the miR-532-5p promoter (Fig. 5d). Furthermore, a decrease in the wild-type miR-532-5p promoter and mut1 promoter luciferase activity was observed in ETS1-overexpressing 786-O cells, but no change was observed in the Mut2-type or Mut1&2-type (Fig. 5e, f). Interestingly, ETS1 staining in RCC tissues was markedly upregulated and directly correlated with a clinical stage but was hardly detectable in normal renal tissues (Fig. 5g). Statistical analyses demonstrated that ETS1 expression was effectively associated with tumour size (*p* = 0.018), T stage (*p* = 0.003), and Fuhrman grade of patients with renal cell carcinoma (*p* = 0.011) (Table S13). Additionally, univariate analysis demonstrated that a higher ETS1 expression level (HR = 1.65; 95% CI = 1.17–2.34; *p* = 0.005), a larger tumour size (HR = 1.49; 95% CI = 1.20–1.83; *p* = 0.018), and a higher clinical T stage (HR = 1.86; 95% CI = 1.31–2.36; *p* = 0.015) were markedly associated with overall survival. Multivariate analysis confirmed that a higher ETS1 expression level (HR = 1.51; 95% CI = 1.05–2.20; *p* = 0.028) and a larger tumour size (HR = 1.53; 95% CI = 1.34–1.97; *p* = 0.036) were substantially correlated with overall survival (Table S14). Moreover, Kaplan–Meier survival analysis from the TCGA RCC dataset showed that RCC patients with low ETS1 expression had longer survival times than patients with high ETS1 expression levels (log-rank test, *p* < 0.05, Fig. 5h, Table S15), in keeping with the same analysis from the Human Protein Atlas (https://www.proteinatlas.org/) (Figure S1H). Taken together, our data suggest that ETS1 silences miR-532-5p transcription by binding to a specific promoter ETS1E VII and could serve as a prognostic marker for RCC.

### KRAS and NAP1L1 are functionally involved in miR-532-5p-suppressed proliferation of RCC cell lines

**Fig. 6:**
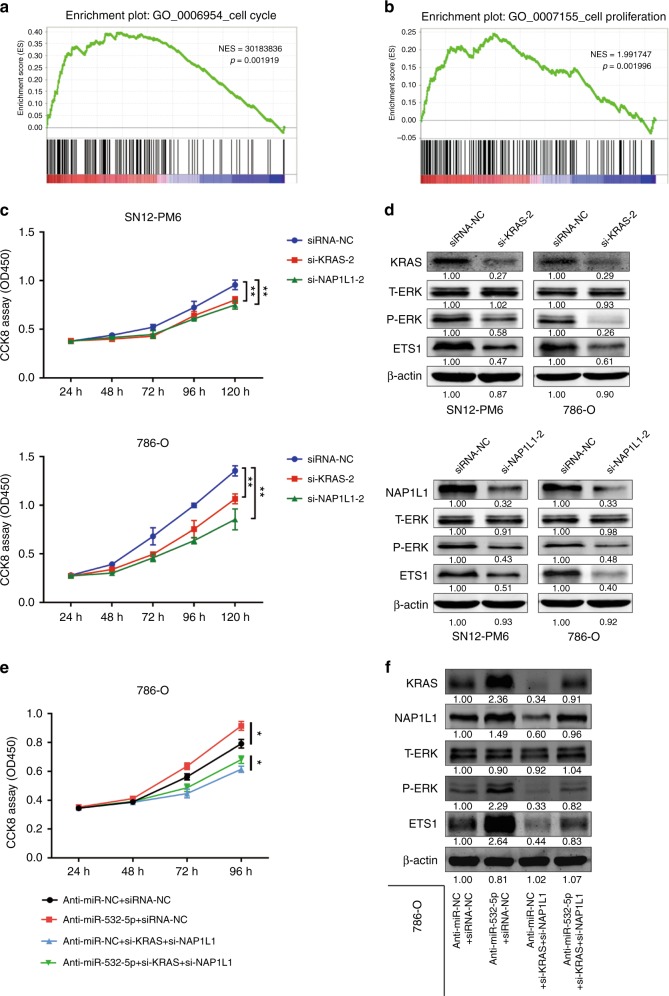
KRAS and NAP1L1 are functionally involved in miR-532-5p-suppressed proliferation of RCC cell lines. **a**, **b** GSEA of the GO_0006954 and GO_0007155 dataset referred to cell cycle and cell proliferation signatures in published miRNA arrays. **c** CCK8 assays of RCC cells transfected with si-KRAS-2 or si-NAP1L1-2 compared to siRNA-NC transfection. The results were averaged from three experiments; error bars indicate ± 1 SD, *p < 0.05, **p < 0.01. **d** Western blot analysis for KRAS, NAP1L1, T-ERK, P-ERK, and ETS1 protein levels of si-KRAS-2 or si-NAP1L1-2 transfection compared to siRNA-NC transfection in SN12-PM6 and 786-O cell lines. β-actin was used as a loading control. **e** CCK8 assays of 786-O cells transfected with anti-miR-NC/anti-miR-53-5p and siRNA-NC/si-KRAS + si-NAP1L1. **f** Western blot analysis for KRAS, NAP1L1, T-ERK, P-ERK, and ETS1 protein levels of 786-O cells transfected with anti-miR-NC/anti-miR-53-5p and siRNA-NC/si-KRAS + si-NAP1L1. β-actin was used as a loading control

To evaluate the biological functions of KRAS and NAP1L1 in RCC, we performed GSEA to link the published gene array analysis to different-stage RCC patient tissues versus matched normal kidney tissue signatures (GEO Datasets: GSE6344; GO_0006954 and GO_0007155). GSEA supported that cell cycle and cell proliferation were significantly enriched in the RCC group, strongly suggesting that RCC is closely related to the cell cycle and cell proliferation (Fig. 6a, b). Next, we picked an siRNA that silenced KRAS and one that silenced NAP1L1 expression at the protein level from two candidates each (Figure S1I). CCK8 assays suggested that si-KRAS-2 or si-NAP1L1-2 retarded cell proliferation, which corresponded to the previous phenotype (Fig. 6c). As expected, WB confirmed that si-KRAS-2 or si-NAP1L1-2 partially reproduced the effect of reduced P-ERK and ETS1 protein expression caused by miR-532-5p in SN12-PM6 and 786-O cells (Fig. 6d). To investigate the combined biological effects of miR-532-5p, KRAS, and ETS1, a CCK8 assay was performed. As shown in Fig. 6e, reduced miR-532-5p expression enhanced the proliferation of 786-O cells. The combination of si-KRAS and si-NAP1L1 (si-KRAS + si-NAP1L1) significantly inhibited the growth capacity of 786-O cells transfected with anti-miR-532-5p. This process was further examined by WB analysis of KRAS, NAP1L1, T-ERK, P-ERK, and ETS1 in 786-O cells. Our results also confirmed that the increase in P-ERK and ETS1 protein levels caused by knockdown of miR-532-5p could be reversed with si-KRAS + si-NAP1L1 (Fig. 6f). In conclusion, the data above suggested that KRAS and NAP1L1 can act as oncoproteins and cause phenotypic alterations in RCC.

### miR-532-5p attenuates tumourigenesis in vivo

**Fig. 7:**
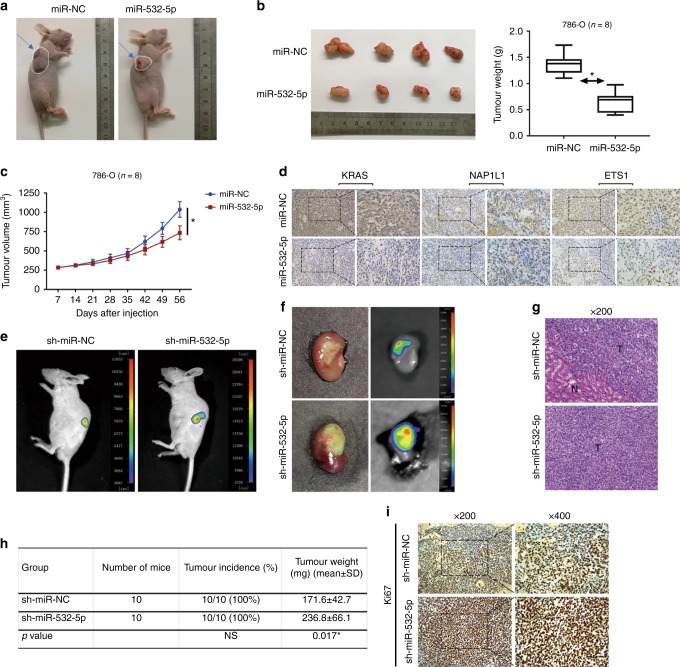
miR-532-5p attenuates tumourigenesis in vivo. **a** The in vivo effect of miR-532-5p was evaluated in subcutaneous xenograft mouse models (*n* = 8) bearing tumours originating from 786-O cells that were stably transfected with miR-532-5p or miR-NC. **b** Macroscopic appearance of the tumours in nude mice from the 8-week-old groups. Weights of the xenografts were shown in the box plot (*n* = 8). **c** Tumour volumes were periodically measured for each mouse and tumour growth curves were plotted. Data represent mean ± S.D. *p < 0.05, **p < 0.01. **d** Representative IHC staining of KRAS, NAP1L1, and ETS1 from the indicated tumours (200×, 400×). **e** Representative images of mice viewed by IVIS bioluminescent imaging system in the sh-miR-532-5p and sh-miR-NC group 4 weeks after injections of the left renal capsule (*n* = 10). **f** Macroscopic appearance and representative images of the tumour xenografts in nude mice viewed by IVIS from the 8-week group with injection of sh-miR-532-5p/sh-miR-NC-SN12-PM6 cells (*n* = 10). **g** H&E (haematoxylin–eosin) staining of the tumour xenografts (200×). **h** Tumour incidence and weights of the xenografts were shown in the table (mean ± S.D.) (*n* = 10). **i** Representative IHC staining of Ki67 from the xenografts (200×, 400×)

To further validate the growth-suppressive function of miR-532-5p, we performed an in vivo tumourigenesis experiment in a xenograft tumour model. In total, 786-O cell lines infected by miR-532-5p-overexpressing lentivirus or miR-NC lentivirus were transplanted subcutaneously into nude mice, and the expression of miR-532-5p, KRAS, and NAP1L1 mRNA or protein levels was confirmed by qRT-PCR and WB (Figure S1J and S1K). As expected, tumour volumes, weights, and growth rates were saliently reduced in tumours derived from miR-532-5p-overexpressing 786-O cells versus those from the miR-NC group (Fig. 7a–c). These tumours also exhibited a decrease in KRAS, NAP1L1, and ETS1 expression in 786-O/miR-532-5p cells, as assessed by IHC (Fig. 7d). Moreover, in vivo studies of mice harboring tumour clones with sh-miR-532-5p or sh-miR-NC were performed. SN12-PM6 cells with firefly luciferase expression were transfected with sh-miR-532-5p or sh-miR-NC, and the expression of miR-532-5p, KRAS, and NAP1L1 mRNA or protein levels was confirmed by qRT-PCR and WB (Figure S1L and S1M). Next, the stable clones were inoculated into the left kidney capsule of nude mice, and tumour growth was measured by IVIS. As shown in Fig. 7e, a dramatic induction in luciferase expression occurred in tumours in the sh-miR-532-5p group as early as the 4th week, indicative of increased tumour proliferation compared with the sh-miR-NC group. Eight weeks after injection, we performed ex vivo bioluminescent imaging immediately after mice were killed to monitor renal tumours. The luciferase expression of tumours from the sh-miR-532-5p group was higher than the expression of tumours from the sh-miR-NC group, which was positively correlated with the tumour size (Fig. 7f, g). The incidence of renal tumours and tumour weight in orthotopic xenografts after 8 weeks was recorded, and tumour weight was calculated by subtracting the weight of the right kidney (normal) from the weight of the left kidney (implanted with tumour) (Fig. 7h). However, there was no difference in tumour incidence between the sh-miR-532-5p and the sh-miR-NC group. Specifically, IHC analysis showed that there were higher Ki-67 levels in tumours from the sh-miR-532-5p group compared with the sh-miR-NC group (Fig. 7i). Taken together, these results supported the notion that miR-532-5p exerts a significant inhibitory effect on tumourigenesis in vivo.

## Discussion

A good deal of evidence has suggested that miR-532-5p is involved in multiple human cancers.^12,13,34,38^ For example, overexpression of miR-532-5p leads to a better prognosis in ovarian cancer.^13^ On the contrary, miRNA-532-5p functions as an oncogenic miRNA in human gastric cancer.^12^ Nevertheless, the biological roles and molecular mechanisms of miR-532-5p, whether it facilitates or abrogates tumour progression in RCC, have not been reported. We first identified that miR-532-5p expression was decreased in RCC tissues and cell lines compared to non-tumour tissues, as well as HK-2 normal cells. Furthermore, bona fide miR-532-5p not only significantly suppressed cell proliferation in vitro, but it also inhibited tumourigenicity in vivo, by targeting the 3’-UTRs of KRAS and NAP1L1, affecting downstream genes, such as P-ERK and ETS1 expression. The biological function of miR-532-5p identified in this study provided a mechanism for its role in anticarcinogenesis. Based on the previous study, which showed that miRNAs interact with different signalling pathways,^9^ we hypothesised that there might be other pathways, in addition to the MAPK pathway in RCC; we will continue to explore other potential signalling pathways in the future. In addition, reduced miR-532-5p expression was positively associated with the survival of RCC patients. It could be a novel potential biomarker for diagnosis and prognosis in RCC. Overall, these results give firm evidence that miR-532-5p could serve as a potential tumour suppressor in RCC.

Early studies revealed that miRNAs have one or more target genes and influence mRNA degradation or translational inhibition.^8,39^ To make a thorough inquiry of the targets of miR-532-5p in RCC, we used different miRNAs and target-predicting software and found 13 candidates participating in cell proliferation. Fortunately, WB and luciferase assays confirmed that KRAS and NAPL1L1 were potential target genes of miR-532-5p. KRAS is a well-known oncogene that is frequently mutated and activated in many cancers.^40,41^ Specifically, in our study, there is an inverse correlation between miR-532-5p and KRAS, and in keeping with the previous discovery in lung adenocarcinoma cells, KRAS is a tumour driver.^42^ Besides, as another target gene of miR-532-5p in our work, NAP1L1 has been shown to regulate DNA replication and chromatin formation, which contributes to various processes.^43^ Previous evidence revealed that NAP1L1 promoted the growth of iPSC and neuroendocrine neoplasms.^24,33^ Besides their own characteristics, their 3’-UTRs can be bound to miRNAs. This common interaction can make two genes part of a gene regulatory network. KRAS and NAP1L1, as targets of miR-532-5p, could induce RCC cell proliferation by enhancing ERK/MAPK signalling and their synergism would merit further investigation in this loop.

It was widely recognised that the transcription factor ETS1 has a dual function, including enhancing or suppressing downstream gene expression.^14,44,45^ For example, ETS1 serves as a coactivator of ATP-induced MUC5AC overexpression in the airway.^46^ However, ETS1 downregulates IL-1β-induced MUC5AC overproduction during airway inflammation.^47^ Similar results that ETS1 could not only upregulate but also downregulate the expression of different miRNAs in lung squamous cell carcinomas have been reported.^14^ Additionally, ETS1 is a novel MAPK-driven transcription factor in squamous cell carcinomas.^14^ In this work, we reported that the protein level of ETS1 was associated with tumour size, T stage, and the Fuhrman grade of RCC patients, which could be a novel potential biomarker in RCC diagnosis and treatment. WB suggested that ETS1 was downregulated through ERK/MAPK signalling in miR-532-5p-overexpressing cells. Conversely, ChIP assays uncovered that ETS1 could inhibit miR-532-5p expression by physically binding the special ETS1-binding element in the miR-532-5p promoter. Taken together, the cross-talk between miR-532-5p, KRAS, NAP1L1, and EST1 established a bridge between miR-532-5p and the MAPK-signalling pathway, demonstrating that miRNAs and their target genes participate in the development and progression of tumours.

**Fig. 8:**
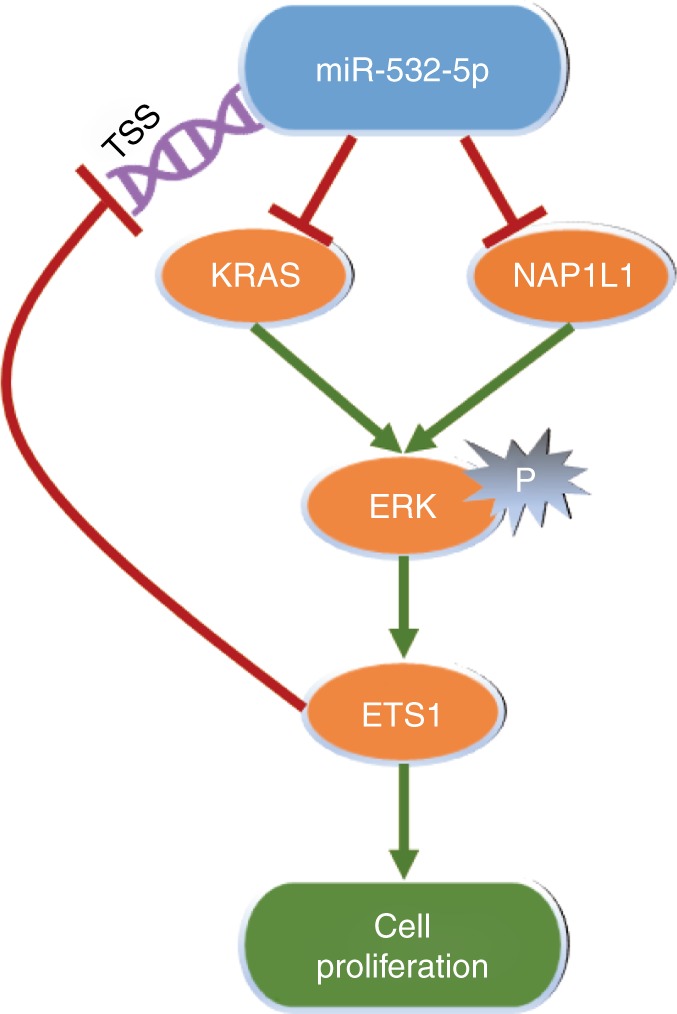
A schematic for an miR-532-5p/KRAS-NAP1L1/P-ERK/ETS1 feedback loop. miR-532-5p downregulated the MAPK-signalling pathway by directly targeting its downstream genes KRAS and NAP1L1. MAPK-signalling pathway in turn downregulated miR-532-5p by ETS1 directly binding to its promoter

As summarised in Fig. 8, miR-532-5p forms a feedback loop through the KRAS-NAP1L1/P-ERK/ETS1 signalling pathway, and it leads to the inhibition of cell proliferation in RCC. Given how frequently the MAPK-signalling pathway plays a vital role in numerous cancer types, our finding has important implications for improved treatment of RCC by regulating miR-532-5p-mediated MAPK signalling. At the same time, there is an increasing appreciation that miRNAs form regulatory networks with protein regulators.^48^ Therefore, our work contributes to our understanding of the role of miRNAs in the regulatory networks involved in RCC and merits further preclinical and prospective clinical validation. Targeting this newly identified feedback loop may help us to better suppress RCC progression.

## Electronic supplementary material

S1-1

S1-2

Supplementary Figure Legends

TS1

TS2

TS3

TS4

TS5

TS6

TS7

TS8

TS9

TS10

TS11

TS12

TS13

TS14

TS15
