# Supplementary figures and images for: MiR-532-5p suppresses renal cancer cell proliferation by disrupting the ETS1-mediated positive feedback loop with the KRAS-NAP1L1/P-ERK axis
Source: Br J Cancer. 2018 Aug 7;119(5):591–604. doi: 10.1038/s41416-018-0196-5 (PMC6162242; doi:10.1038/s41416-018-0196-5)

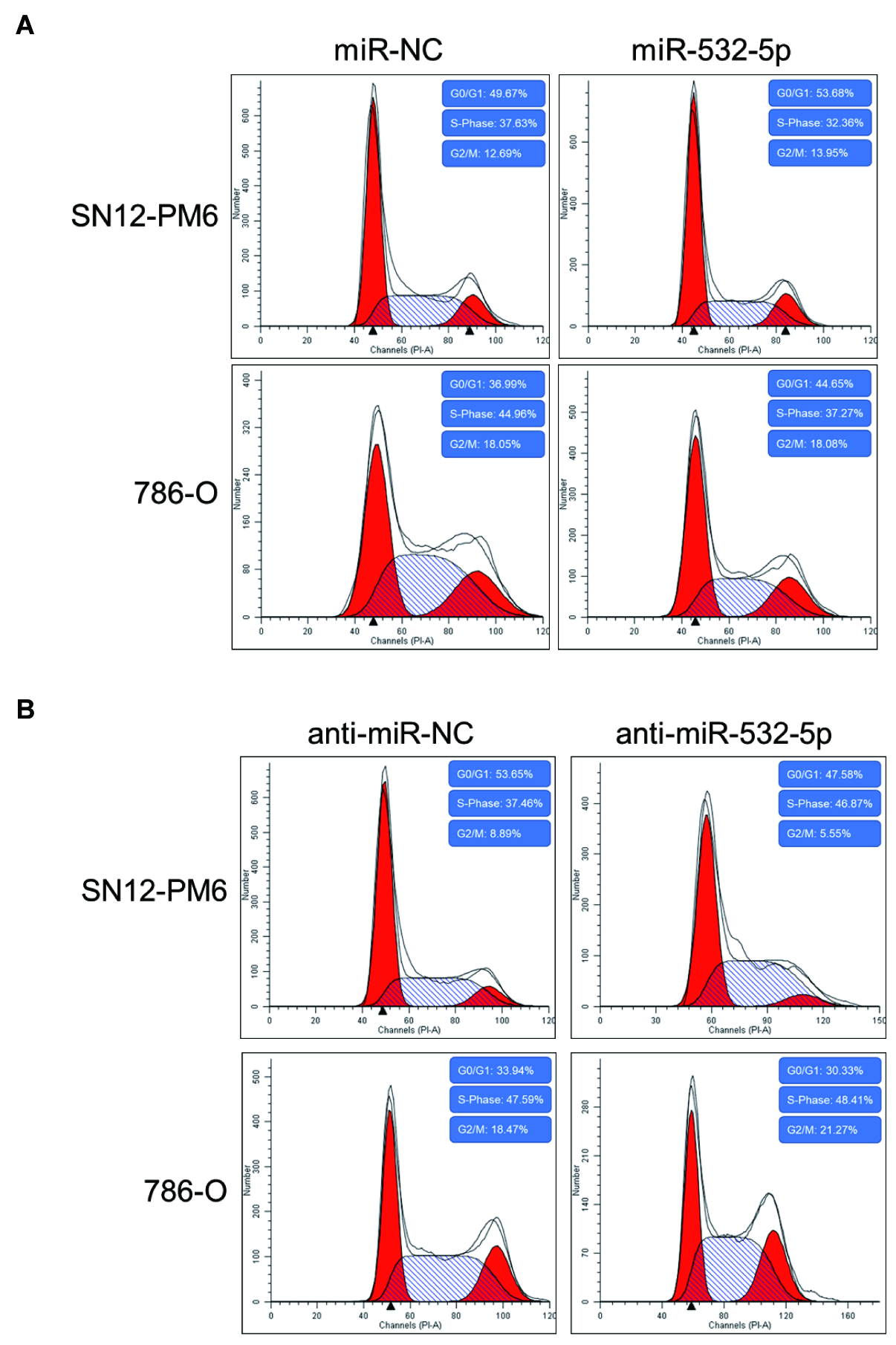

Supplement: Supplementary file 1 — S1-1 [file 41416_2018_196_MOESM1_ESM.tif]

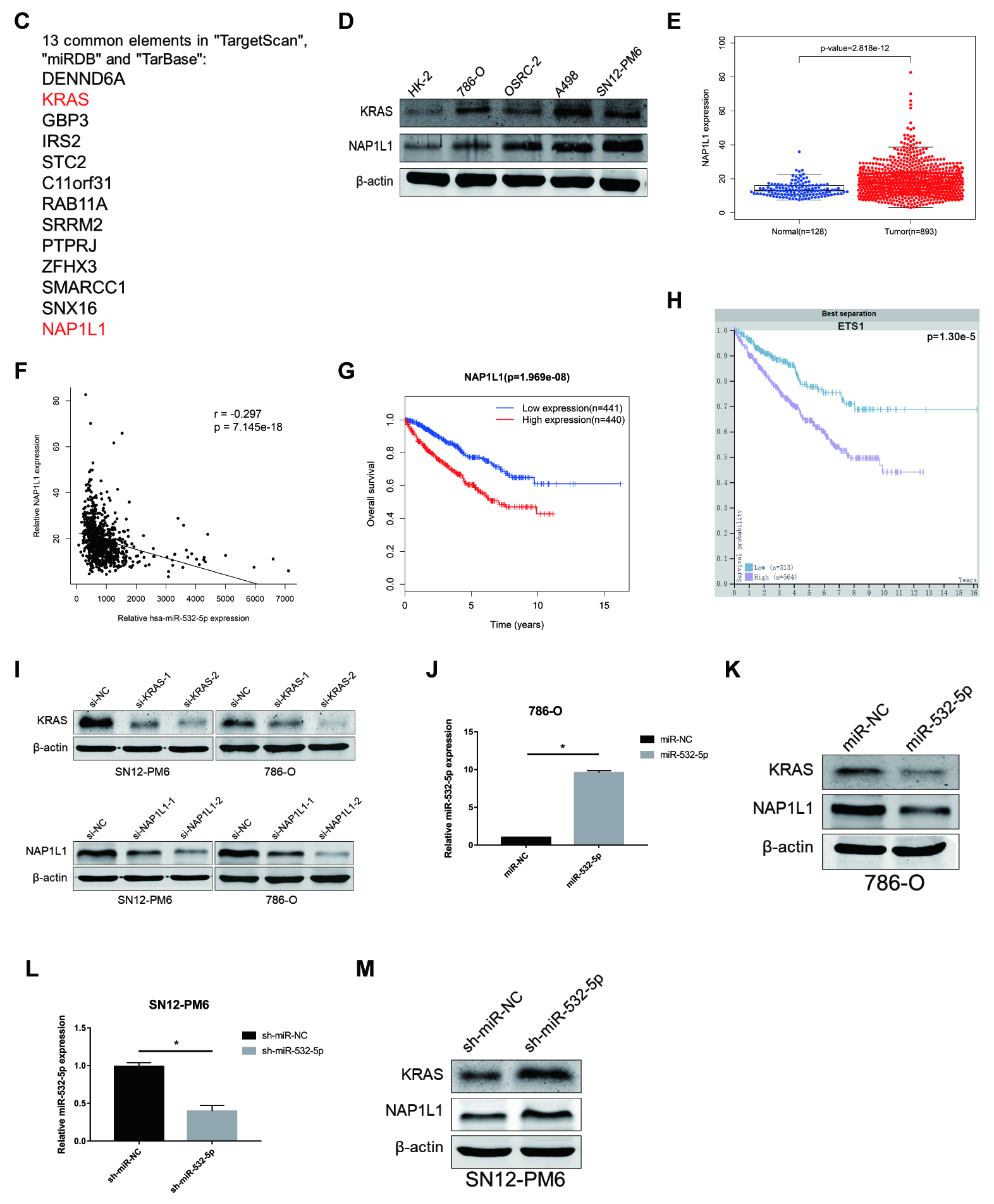

Supplement: Supplementary file 2 — S1-2 [file 41416_2018_196_MOESM2_ESM.tif]
