# Supplementary material for: MiR-532-5p suppresses renal cancer cell proliferation by disrupting the ETS1-mediated positive feedback loop with the KRAS-NAP1L1/P-ERK axis
Source: Br J Cancer. 2018 Aug 7;119(5):591–604. doi: 10.1038/s41416-018-0196-5 (PMC6162242; doi:10.1038/s41416-018-0196-5)
Supplement: Supplementary file 3 — Supplementary Figure Legends [file 41416_2018_196_MOESM3_ESM.docx]

**Supplementary Figure Legends**

**Supplementary Figure 1.**

**A:** The representative results of cell cycle analysis by flow cytometer in SN12-PM6 and 786-O cells transfected with miR-NC and miR-532-5p mimic. **B:** The representative results of cell cycle analysis by flow cytometer in SN12-PM6 and 786-O cells transfected with anti-miR-NC and anti-miR-532-5p. **C**: 13 predicted target genes in "TargetScan", "miRDB" and "TarBase". **D:** The expression of KRAS and NAP1L1 were determined in several human RCC cell lines (786-O, OSRC-2, A498, SN12-PM6) and human normal renal tubular epithelial cell line HK-2 by WB. β-actin was used as a loading control. **E:** NAP1L1 expression in RCC and normal samples from TCGA RCC dataset. **F:** TCGA RCC dataset indicated the correlation between NAP1L1 and miR-532-5p in RCC. **G:** Kaplan–Meier analyses of the correlations between NAP1L1 expression and overall survival of 881 RCC patients from TCGA RCC dataset. Log-rank test was used to calculate p values. **H:** Kaplan–Meier analyses of the correlations between ETS1 expression and overall survival of 877 RCC patients from THE HUMAN PROTEIN ATLAS (https://www.proteinatlas.org/). Log-rank test was used to calculate p values. **I:** WB analysis for KRAS or NAP1L1 protein levels after the transfection with si-KRAS (si-KRAS-1 and si-KRAS-2) or si-NAP1L1 (si-NAP1L1-1 and si-NAP1L1-2)， and siRNA-NC in SN12-PM6 and 786-O cells. β-actin was used as a loading control. **J:** The expression of miR-532-5p was determined by qRT-PCR in 786-O cells after the infection with miR-532-5p or miR-NC lentivirus. **K:** WB analysis for KRAS and NAP1L1 protein levels after the infection with miR-532-5p or miR-NC lentivirus. β-actin was used as a loading control. **L:** The expression of miR-532-5p was determined by qRT-PCR in SN12-PM6 cells after the infection with sh-miR-532-5p or sh-miR-NC lentivirus. **M:** WB analysis for KRAS and NAP1L1 protein levels after the infection with sh-miR-532-5p or sh-miR-NC lentivirus. β-actin was used as a loading control.
